# Prosthesis-Patient Mismatch Following Aortic Valve Replacement: Finding Predictors for Prevention

**DOI:** 10.36660/abc.201907

**Published:** 2020-01

**Authors:** Flávio Tarasoutchi

**Affiliations:** Instituto do Coração (InCor) - Faculdade de Medicina da Universidade de São Paulo, São Paulo, SP - Brazil

**Keywords:** Aortic Valve/surgery, Heart Valve Prosthesis/adverse effects, Size Perception, Quality of Life, Echocardiography/methods

Mismatch between the size of an aortic bioprosthesis and the patient (prosthesis-patient mismatch - PPM) is a poorly studied entity, and can be associated with adverse postoperative results, with quality of life impairment and a worse prognosis among those with severe PPM.^1^

## The impact of prosthesis-patient mismatch

Nowadays, the main indication for valve surgery, in patients with anatomically important aortic valve disease, occurs at the onset of symptoms, given the presumed benefit of reduced morbidity and mortality associated with this procedure in this context.^2-4^ Thus, the presence of PPM following surgical implantation of an aortic bioprosthesis interferes in the expected reduction of symptomatology and mortality rates, thus minimizing the benefits that such invasive procedure could bring to the patient.^5^ Therefore, we need tools to predict the risk of PPM and thus implement strategies that can prevent such entity. This scenario was evaluated by Otto et al.^6^ in the current issue of the *Arquivos Brasileiros de Cardiologia*.

PPM is defined echocardiographically by an indexed effective orifice area (EOA) ≤ 0.85cm^2^/m^2^ and considered severe if ≤ 0.65 cm^2^/m^2^.^7^ Since this parameter is indexed by body surface area, individuals with body mass index ≥ 35 kg/m^2^ have lower reference values (≤ 0.70 cm^2^/m^2^ and ≤ 0.55 cm^2^/m^2^, respectively) to avoid overestimation of anatomical severity in these patients.^8^ Its reported prevalence varies greatly: up to 70% in the case of moderate PPM and 20% in the case of severe PPM.^5^

## The current study

In this study, the authors showed a 33.4% incidence of severe PPM in a representative population treated by the Brazilian Unified Health System (SUS), which is significantly greater than that described in other studies.^5^ This fact can be justified by the study design, but also because it deals with a characteristically different population, with prevalence of young patients and with rheumatic etiology.

Furthermore, the authors created a model for prediction of severe PPM, containing the following parameters: age, male sex, LV outflow tract diameter ≤ 2.1 cm, body mass index and etiology of valve disease. A specific score for this prediction, composed by preoperative factors, is extremely relevant to identify patients in need for differential interventions and to avoid PPM. In elderly patients with degenerative aortic stenosis, a transcatheter aortic valve implantation (TAVI) may be beneficial. There is evidence that the incidence of PPM in patients who underwent TAVI is lower compared with patients undergoing conventional surgery, especially among those with a small aortic annulus.^9^ Currently, to test this hypothesis, there is a multicenter randomized trial (Transcatheter Aortic Valve Replacement Versus Surgical Aortic Valve Replacement for Treating Elderly Patients With Severe Aortic Stenosis and Small Aortic Annuli: A Prospective Randomized Study - The VIVA Trial; NCT03383445) comparing TAVI and conventional surgery in elderly patients with a small aortic annulus (average diameter of the aortic annulus < 22 mm).^9^

On the other hand, for young patients with rheumatic disease, like those in this study, there are surgical therapeutic alternatives, such as aortic annulus enlargement, implantation of supra-annular prostheses, sutureless prostheses and stentless prostheses. However, the literature is still scarce on this issue and randomized studies are expected to determine the best treatment among them.^10^

This study has some limitations. The retrospective cross-sectional design and the exclusion of about half of the initial population due to data loss may have resulted in overestimation of the prevalence of PPM, which reiterates the need for prospective studies on the issue.

## Conclusion

Predicting PPM is important and continues to be a dilemma. The study carried out by Otto et al.^6^ brings relevant information on this entity in a selected population of the Brazilian Unified Health System (SUS). New prospective studies are needed for a better understanding of PPM and also for validation of the proposed score.
